# Pituitary Stalk Interruption Syndrome

**DOI:** 10.7759/cureus.10518

**Published:** 2020-09-17

**Authors:** Tehreem Fatima, Sajjad Hussain Chandio, Kainat Muzaffar, Hassan Mumtaz, Nusrat Jahan

**Affiliations:** 1 Internal Medicine, California Institute of Behavioral Neurosciences & Psychology, Fairfield, USA; 2 Gastroenterology, Holy Family Hospital, Rawalpindi, PAK; 3 Internal Medicine, Holy Family Hospital, Islamabad, PAK; 4 Urology, Guy’s and St Thomas' NHS Foundation Trust, London, GBR; 5 General Medicine, Surrey Docks Health Centre, London, GBR; 6 Internal Medicine, Holy Family Hospital/Rawalpindi Medical University, Rawalpindi, PAK

**Keywords:** psis, pituitary stalk interruption syndrome, growth hormone deficiency

## Abstract

Pituitary stalk interruption syndrome (PSIS) is a rare, congenital disorder characterized by a triad of a thin or interrupted pituitary stalk, aplasia or hypoplasia of the anterior pituitary, and absent or ectopic posterior pituitary (EPP) seen on magnetic resonance imaging (MRI). It can either present at birth or later in life. PSIS is very heterogeneous with respect to its hormonal, clinical, and radiological presentation. The patient described is an 18-year-old male who presented with complaints of short stature and underdeveloped secondary sexual characteristics with weight and height of less than three percentiles for his age. The secondary sexual characteristics were consistent with Tanner Stage II. Some of the pituitary hormones were also deficient. Wrist X-ray was compatible with a bone age of a seven- to eight-year-old. MRI confirmed the diagnosis of PSIS (pituitary stalk interruption syndrome).

## Introduction

Pituitary stalk interruption syndrome (PSIS) is a rare congenital disorder with a reported incidence of 0.5/100,000 live births [1]. The first case was reported in 1987 by Fujisawa et al. after surgical resection of pituitary stalk in patients with idiopathic pituitary dwarfism [2]. It is characterized by a triad of thin (<1 mm) or interrupted pituitary stalk, hypoplasia, or aplasia of the anterior lobe and with the presence of an ectopic posterior pituitary (EPP) [3]. It is heterogeneous regarding its clinical, hormonal, and radiological presentation. Furthermore, age at onset is highly variable as well. The diagnosis is confirmed through magnetic resonance imaging (MRI). Improved MRI performance over recent years has allowed the identification of this condition. Two typical PSIS clinical manifestations have been described that include anterior pituitary hormone deficiencies either at birth or during the pediatric age. The PSIS that presents later in life appears gradually and generally progresses in adulthood to panhypopituitarism [4].

Our case presented later in life.

## Case presentation

Here we present a case of an 18-year-old male patient with complaints of short stature and underdevelopment of secondary sexual characteristics.

Birth history was insignificant, and he was born at full term without complications. His birth weight was 3 kg, and his family history was unremarkable. The patient sometimes complained of lethargy and generalized weakness but was treated symptomatically in the past. There was no history of delayed achievement of developmental milestones and the patient had a good academic record. During childhood, he had all his vaccinations and had not been hospitalized. There was no known drug allergy or history of smoking. He was a non-diabetic and non-hypertensive and had no other known comorbidities. History of previous surgeries and previous hospitalizations was also insignificant.

On examination, the patient was a young man of an average build without any abnormal facial features, who was fully aware of time, space, and person. He had short stature and underdeveloped secondary sexual characteristics with decreased facial, pubic, and axillary hair growth. His weight was 20 kg and height 100 cm (body mass index [BMI] 20 kg/m2). Weight and height were less than third percentiles for age. Testicular volume was 3 ml each, measured by using a Prader orchidometer. Pubic hair distribution and penile size were both consistent with Tanner Stage II. No gynecomastia was noted. Heart and breath sounds were normal and so was the abdominal examination.

**Figure 1:**
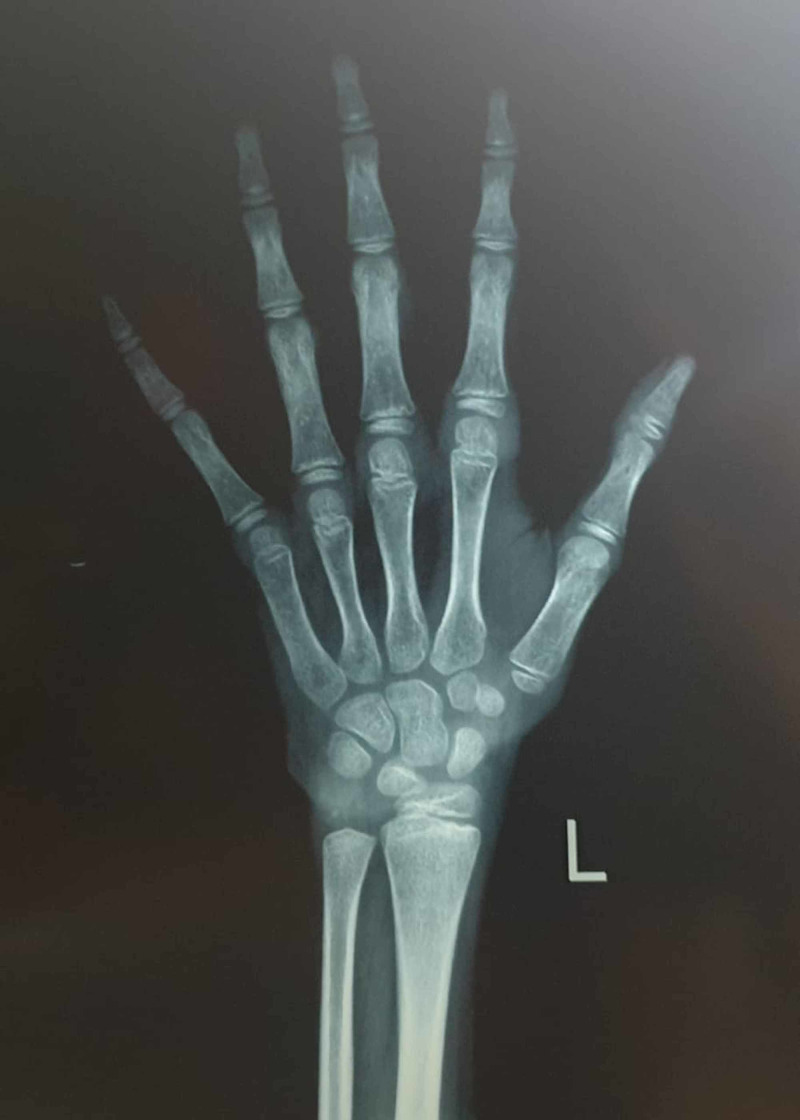
X-Ray of wrist to assess bone age of the patient. The bone age of trapezium, trapezoid, and scaphoid appears to be six years and the bone age of the distal epiphysis of the ulna seems to be five to eight years. That makes approximate bone age to be around seven to eight years. The chronological age of the patient is 18 years.

Routine blood panel and urine and stool tests were within the normal range, except for mild anemia. A growth hormone (GH) stimulation test was consistent with GH deficiency. Other pituitary hormones were also evaluated and revealed adrenal insufficiency. Surprisingly, there was no central hypothyroidism. X-Ray films of the wrist were ordered and were compatible with a bone age of a seven- to eight-year-old (>2 SD below chronological age) as shown in Figure 1.

As part of the investigation, magnetic resonance imaging (MRI) brain with and without contrast injection was performed. The sagittal T1-weighted image before and after the contrast injection showed a relatively small anterior pituitary, ectopic posterior pituitary, and absent infundibular stalk. A well-defined nodule noted in the median eminence in the tuber cinereum of the floor of the third ventricle measuring 2.3 x 3.8 x 2.8 dimensions. It appears high on T1 and T2 weighted images and suppressed on Fat-Sat (Fat Saturation pulses are short duration radiofrequency pulses to null the signal from fat tissue) which together with the absence of normally located bright signals of the neurohypophysis and the pituitary stalk on the sagittal images, was indicative of ectopic posterior pituitary (EPP).

The MRI findings were pointing towards the diagnosis of pituitary stalk interruption syndrome (PSIS). Based on the clinical and radiological findings, the diagnosis was made, and hormone replacement therapy was started.

## Discussion

PSIS is a rare clinical entity with an incidence of 0.5/100,000. Lately, more cases of PSIS are being recognized, after the use of MRI as a primary radiological modality in patients with panhypopituitarism. The mean age at the time of diagnosis is 9.4 ± 11.6 years. Interestingly, male predominance has also been reported with a male-to-female ratio of 2.3-6.9:1.0, suggesting X-linked inheritance [1]. The incidence of breech delivery, cesarean section, and neonatal distress are high in the PSIS population [5]. Breech delivery leads to deformation of the head which can result in injury to the pituitary stalk. Similarly, hypoxemia due to anoxia after birth may also cause injury of the pituitary stalk and pituitary [5]. Genetic mutations are thought to contribute to less than 5% of PSIS cases. Mutations detected were in HESX1, LHX4, SOX3, PROKR2, and OTX2 genes [6].

Clinical presentations

Due to the limited number of reported cases of PSIS, the clinical manifestations of this disease are diverse. The severity of the hormone deficiency determines the age of diagnosis. When PSIS occurs at birth, hypoglycemia, and failure to thrive are the most common symptoms. In childhood, growth retardation is typically the main complaint, while delayed puberty is usually the main concern when it manifests in adolescence and early adulthood [6]. These patients have multiple hormonal deficiencies. Growth hormone deficiency (GHD) is the most frequently existing deficiency and reaches 100% of all patients. Interestingly, considerable heterogeneity in height has been reported. Few children maintain normal linear growth despite abnormal GH secretion [4]. Gonadotropin deficiency (luteinizing hormone [LH], follicle-stimulating hormone [FSH]) is frequently associated with other deficiencies. Adrenocorticotropic hormone (ACTH) deficiency can cause neonatal cholestasis and recurrent hyponatremia. Stimulation test for ACTH and cortisol measurement have shown their levels to be significantly lower in patients with PSIS [4]. Prolactin (PRL) levels have shown a considerable degree of heterogeneity. It can be deficient, or on the opposite hyperprolactinemia can be observed, from 17% to one-third of patients. It varies depending on the severity of dopaminergic pathway disconnection [4]. Owing to different embryonic origins of the anterior and posterior pituitary, only a few patients have complained of central diabetes insipidus. In the neonatal period, features indicative of hypopituitarism; hypotonia, secondary adrenal deficiency with hypotension, and prolonged cholestatic icterus, repeated episodes of hypoglycemia are found in 33% of PSIS patients [4].

Thyroid-stimulating hormone (TSH) deficiency may also be found but measurement may be within the normal limits in most patients with central hypothyroidism. Cases have been described with isolated sparing of TSH secretion with a deficiency of the remaining anterior pituitary hormones [4]. Our patient’s thyroid function tests favored secondary hypothyroidism because of a very low free T4 (FT4) level and slightly increased TSH level, together with a negative anti-thyroid antibody level; Similar findings were also reported by Guo et al. in a subgroup of PSIS patients [5]. Interestingly, patients initially presenting with isolated GH deficiency initially, may develop multiple anterior pituitary hormone deficiencies later in life in the second or third decade of life [1]. Hence, a close follow-up of PSIS patients is required.

MRI is key for PSIS investigations. PSIS has a heterogeneous radiological presentation due to the multiple etiological mechanisms. Imaging has shown that the anterior lobe can be absent, hypoplastic or normal and the pituitary lobe can be absent or ectopic; along the stalk or at the hypothalamic base, etc. There is a lot of variation involving the stalk as well. It could be interrupted, thin, or normal [4].

Treatment is based on substituting hormones with life-long poly-hormonal replacement therapy.

## Conclusions

Pituitary hormone deficiency in PSIS leads to long-term morbidities. Early identification of missing hormones and hormonal replacement influences both the prognosis and the quality of life in patients with hypopituitarism. If PSIS patients present before the joining of epiphyses, they have an excellent opportunity to reach their normal height and prevent short stature. A regular follow-up is required as some hormone deficiencies can develop later in life.
